# Quantitative analysis of chest MRI images for benign malignant diagnosis of pulmonary solid nodules

**DOI:** 10.3389/fonc.2023.1212608

**Published:** 2023-08-04

**Authors:** Bin Yang, Yeqi Gao, Jie Lu, Yefu Wang, Ren Wu, Jie Shen, Jialiang Ren, Feiyun Wu, Hai Xu

**Affiliations:** ^1^Department of Radiology, The First Affiliated Hospital of Nanjing Medical University, Nanjing, China; ^2^Department of Medical Imaging, Jinling Hospital, Nanjing Medical University, Nanjing, China; ^3^Department of Pharmaceuticals Diagnostics, GE Healthcare, Beijing, China

**Keywords:** magnetic resonance imaging, radiomics, machine learning, pulmonary nodules, differential diagnosis

## Abstract

**Background:**

In this study, we developed and validated machine learning (ML) models by combining radiomic features extracted from magnetic resonance imaging (MRI) with clinicopathological factors to assess pulmonary nodule classification for benign malignant diagnosis.

**Methods:**

A total of 333 consecutive patients with pulmonary nodules (233 in the training cohort and 100 in the validation cohort) were enrolled. A total of 2,824 radiomic features were extracted from the MRI images (CE T1w and T2w). Logistic regression (LR), Naïve Bayes (NB), support vector machine (SVM), random forest (RF), and extreme gradient boosting (XGBoost) classifiers were used to build the predictive models, and a radiomics score (Rad-score) was obtained for each patient after applying the best prediction model. Clinical factors and Rad-scores were used jointly to build a nomogram model based on multivariate logistic regression analysis, and the diagnostic performance of the five prediction models was evaluated using the area under the receiver operating characteristic curve (AUC).

**Results:**

A total of 161 women (48.35%) and 172 men (51.65%) with pulmonary nodules were enrolled. Six important features were selected from the 2,145 radiomic features extracted from CE T1w and T2w images. The XGBoost classifier model achieved the highest discrimination performance with AUCs of 0.901, 0.906, and 0.851 in the training, validation, and test cohorts, respectively. The nomogram model improved the performance with AUC values of 0.918, 0.912, and 0.877 in the training, validation, and test cohorts, respectively.

**Conclusion:**

MRI radiomic ML models demonstrated good nodule classification performance with XGBoost, which was superior to that of the other four models. The nomogram model achieved higher performance with the addition of clinical information.

## Background

Lung cancer is one of the most common malignant neoplastic diseases worldwide, and its mortality rate is the highest in the world, with incidences second only to breast cancer (1). According to the latest national cancer statistics data released by the National Cancer Center in 2022, in China, the incidence and mortality rate of lung cancer are the highest among neoplastic diseases, which seriously threaten the health of the Chinese population (2). The 5-year survival rate for lung cancer remains less than 18% due to the lack of symptoms in the early stage, late stage when a diagnosis is made, and heterogeneity of the tumor, which influences the efficacy of treatment (3, 4). The 5-year survival rate of patients with stage 0–IA lung cancer (small lung cancer) less than 2 cm in diameter is approximately 100%. Therefore, early diagnosis, accurate staging, and appropriate treatment are important to improve the survival rate of patients with lung cancer. With the wide application of low-dose computed tomography (LDCT) in the screening of pulmonary nodules and the enhancement of people’s health consciousness, the detection rate of early lung cancer has greatly increased, and lung cancer-related mortality has been reduced by approximately 20% (5, 6). High-resolution computed tomography (HRCT) has a sensitivity and specificity of >90% for the diagnosis of benign and malignant lesions in pure ground-glass nodules with a diameter of >6 mm, as well as in some solid nodules (7). However, HRCT diagnosis of solid pulmonary nodules less than 2 cm in diameter only depends on the shape, density, and blood supply; in particular, inflammatory lung cancer and inflammatory granuloma are two diseases that often lead to clinical misdiagnosis and incorrect treatment. PET/CT, a common differential diagnostic method for pulmonary nodules, is widely used in clinical practice. The sensitivity and specificity of PET/CT are 92%–95% and 72%–83%, respectively, because most of the lesions are benign (approximately 60%–70%), such as tuberculosis, fungal infections, and vascular and congenital malformations, with strong glucose metabolism, resulting in high false-positive rates (8). In addition, transthoracic CT-guided needle biopsy of pulmonary nodules is an additional means of obtaining a benign or malignant diagnosis, with a sensitivity of approximately 81%–97% for the diagnosis of lung cancer, but it is invasive, with a high incidence of pneumothorax complications of up to 15% (9).Therefore, it has the ability to differentiate between benign and malignant solid nodules less than 2 cm in diameter, avoid unnecessary invasive examinations and surgical trauma, and prevent tumor progression due to follow-up. At present, it is a hot point for doctors in the imaging, thoracic surgery, and respiratory departments to treat these types of lung cancer patients in a timely and effective manner.

With the rapid development of magnetic resonance imaging (MRI) hardware and software, the imaging speed and signal-to-noise ratio of images have improved, and there is no harm caused by ionizing radiation. MRI-DWI can be used to obtain diffusion-weighted imaging (DWI), apparent diffusion coefficient (ADC) images, and ADC values of quantitative indices. Quantitative indices of tissue and lesion diffusion can be obtained, providing a new examination method for the differential diagnosis of benign and malignant pulmonary lesions. It is helpful to accurately diagnose necrosis, hemorrhage, fat, hilar and mediastinal lymph node metastasis in pulmonary nodules, the deficiency of CT examination was made up. With the development of DWI and dynamic contrast-enhanced imaging (DCE) based on traditional MRI techniques (10, 11), various metabolic and pathological changes in living tissues can be observed noninvasively in the early stages, which endows the functional imaging capability of pulmonary nodules and plays a key role in differentiating benign from malignant pulmonary nodules. Studies have shown that (12–14) using the b-value and ADC of DWI techniques has a sensitivity of 70%–89% and a specificity of 61%–97% for the diagnosis of benign and malignant pulmonary nodules. Perfusion dynamics based on the DCE technique plays a central role in distinguishing benign from malignant solid nodules. The DCE technique can obtain a large amount of parameter information through time-signal intensity curves, and the main curve of malignant nodules is characterized by an early peak followed by a rapid decline (15). Studies have shown that the DCE technique has a sensitivity of 52%–100%, specificity of 52%–96%, and diagnostic accuracy of 75%–94% for differentiating benign from malignant solid nodules (16–18). In addition, it has been found that DWI and DCE, two key techniques in high-resolution MRI of the lung, have synergistic effects in the diagnosis of solid pulmonary nodules less than 2 cm in diameter; however, there are few relevant studies, and the limitations of MRI in the diagnosis of benign and malignant pulmonary nodules are that the sample size is generally small, the diagnostic efficacy, sensitivity, specificity, and accuracy are not high, and its stability is lacking (12, 19, 20).

Artificial Intelligence (AI) technology has shown incomparable advantages in mining medical image information. It is expected that the key imaging markers and dominant features of solid small pulmonary nodules (<2 cm) can be extracted from multisequence MRI images for early diagnosis, treatment decisions, and prognosis evaluation of lung cancer. Recently, radiomics has become a new technology in the field of medical imaging. It can extract large amounts of high-throughput quantitative information, combine clinical data, and build prediction models using machine learning. Ultimately, it is used to guide clinical decision-making. It has been widely used in lesion detection, disease diagnosis, classification, treatment planning, and prognostic scenarios for various diseases, with extremely high application value (21). However, most radiomics methods used for the identification of benign and malignant solid pulmonary nodules are based on CT and PET/CT, and MRI-based radiomics has been used to identify benign and malignant solid lung nodules with a small sample size, poor diagnostic efficacy, and a lack of external and prospective verification (22, 23).

Therefore, this study aimed to construct a predictive model based on the imaging label of MRI and clinicopathologic features to achieve early, sensitive, and accurate diagnosis of pulmonary nodules, improve the accuracy of diagnosis and the survival rate of lung cancer patients, and provide technical support for the accurate treatment of pulmonary nodules.

## Methods

### Patients and clinicopathological data

**Figure 1:**
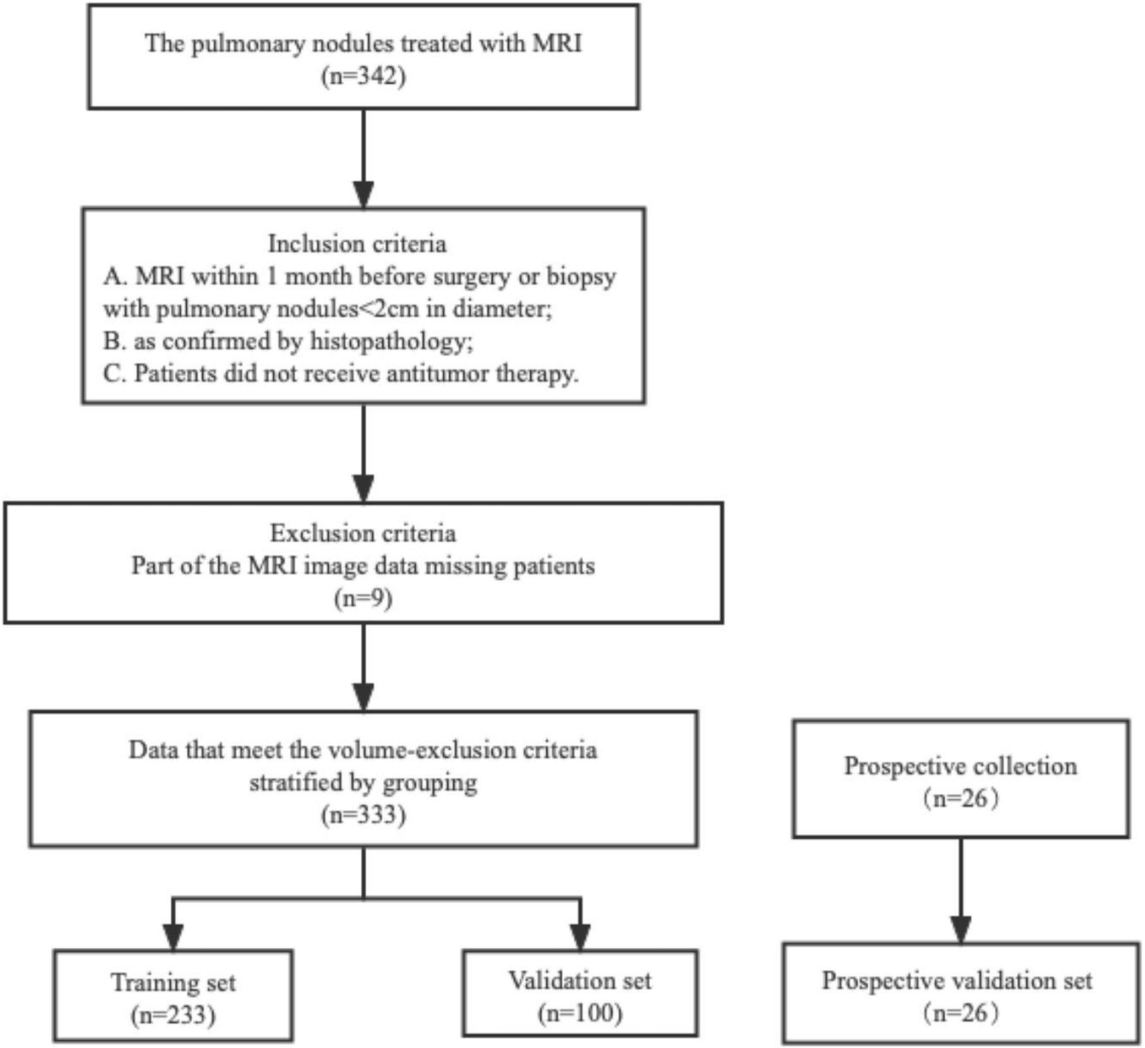
The patient stowage flowchart was divided into training and validation sets in a ratio of 2:1.

This study was conducted at the First Affiliated Hospital of Nanjing Medical University and approved by the Institutional Review Board in accordance with the ethical standards of the 1964 Helsinki Declaration and its subsequent amendments. Informed consent was not required, as protected health information (PHI) was not disclosed. This retrospective study reviewed the medical records of patients between August 2019 and May 2021, focusing on 342 patients with lung nodules (<2 cm) confirmed through histopathological analysis and follow-up. Additionally, 26 patients were prospectively enrolled. To ensure patient privacy, personal information was anonymized prior to data analysis. The study protocol was approved by the Institutional Review Board at the First Affiliated Hospital of Nanjing Medical University (2022-NT-11, Nanjing, China).Patients with the following characteristics were included in the study: (a) patients undergoing MRI examination within 1 month before surgery or biopsy, (b) patients who did not receive antitumor treatment before MRI examination, and (c) patients with histologically confirmed non-small cell lung cancer (NSCLC) through surgery or biopsy. However, patients with the following characteristics were excluded: (a) partial loss of MRI images (n = 9), (b) diseases not related to NSCLC (n = 0), and (c) unclear tumor boundaries that could not be accurately delineated (n = 0). The final cohort included 333 patients (**Figure 1**). A total of 92 cases of malignant nodules were identified, with 69 cases confirmed by surgical pathology and 23 cases confirmed by CT-guided puncture biopsy pathology. Among 92 cases of malignant nodules, the pathological subtypes were 61 adenocarcinoma and four squamous cell carcinomas, not otherwise specified (NOS) 27, respectively (**Table S1**). Additionally, 241 cases of benign nodules were observed, with 57 confirmed by surgical pathology, and 27 confirmed by CT-guided puncture biopsy pathology. In addition, the lesions did not increase after a follow-up interval of 3–6 months. One hundred fifty-seven cases underwent anti-inflammatory treatment, and follow-up examinations revealed that the lesions had become smaller or disappeared, confirming that they were benign nodules. We randomly divided the patients into a training cohort (n = 233) and a validation cohort (n = 100) in a 2:1 ratio, and 26 patients were prospectively enrolled as the validation cohort. Data on age, sex, smoking history, and family history were also collected (**Table 1**).

**Table 1:** Clinical characteristics of enrolled patients.

Characteristics	Training Cohort	Validation Cohort	Test Cohort	*p*-value
	*N = 233*	*N = 100*	*N = 26*	
Label-N (%)				0.496
benign	169 (72.532%)	72 (72.000%)	16 (61.538%)	
malignant	64 (27.468%)	28 (28.000%)	10 (38.462%)	
Gender-N(%)				0.013
Female	106 (45.494%)	55 (55.000%)	6 (23.077%)	
Male	127 (54.506%)	45 (45.000%)	20 (76.923%)	
Age- (years), Median [Q1;Q3]	54.000 [43.000;64.000]	54.000 [44.000;62.250]	56.500 [43.750;65.500]	0.632
Smoke history-N (%)				0.033
No	70 (65.421%)	40 (83.333%)	10 (55.556%)	
Yes	37 (34.579%)	8 (16.667%)	8 (44.444%)	
Family history-N (%)				0.724
No	102 (95.327%)	47 (97.917%)	17 (94.444%)	
Yes	5 (4.673%)	1 (2.083%)	1 (5.556%)	
Max diameter-(cm), Median [Q1;Q3]	1.200 [0.900;1.600]	1.200 [1.000;1.500]	1.400 [1.300;1.625]	0.302

### MRI image acquisition and analysis

All MRI scans were performed using a 3.0 T MR scanner (Verio Tim; Siemens Medical System, Erlangen, Germany) with a 16-channel torso coil. Conventional imaging protocols included unenhanced axial T1-weighted imaging with repetition time [TR]/echo time [TE] of 140/2.5 ms and axial free-breathing BLADE T2-weighted imaging (TR/TE, 1,200/93 ms). Axial DCE-MRI was performed using a T1-weighed volumetric interpolated breath-hold examination (VIBE) with a radial acquisition trajectory (StarVIBE). Gadodiamide (GE HealthCare, Shanghai, China) was intravenously bolus injected via a power injector at a rate of 4.0 mL/s at a dose of 0.1 mmol/kg, followed by a 20-mL bolus of saline administered at the same injection rate. The acquisition consisted of four baselines and 31 contrast-enhanced images. The temporal resolution was 8.8 s, and the total acquisition time was 5 min and 33 s. The other detailed imaging parameters were as follows: TR ms/TE ms, 3.19/1.13 ms; slice thickness, 3 mm; FOV, 400 mm2; matrix, 160 × 224; and flip angle, 15°. Another contrast-enhanced T1-weighted image was obtained after the completion of DCE MR.

### Tumor segmentation and radiomics feature extraction

This study adhered to the Image Biomarker Standardization Initiative (IBSI) guidelines, and the radiomics prototype software program (Radiomics, Frontier, Siemens) was IBSI-compliant. A volume of interest was drawn semi-automatically around the tumor by a chest radiologist (YB, 10 years of experience) in lung diagnosis using radiomics and confirmed by another chest radiologist (WR,4 years of experience). Both radiologists were blinded to the patients’ clinical information. First, we imported contrast-enhanced T1-weighted (CE T1w) and T2-weighted (T2w) images into Radiomics prototype software (Radiomics, Frontier, Siemens). In the segmentation module of radiomics, a few segmentation tools are available for the semi-automatic delineation of the tumor in three dimensions. Segmentation is produced semi-automatically by drawing a line across the tumor boundary. Then, using an automatic algorithm, the tool finds neighboring voxels with the same gray level in three-dimensional (3D) space, generating random walker-based lesion segmentation for solid and subsolid lung lesions (24). If the segmentation is incorrect, operators can manually correct it in the 3D domain using the radiomics prototype. Before feature extraction, all the MR images were resampled to an isotropic voxel size of 1.00 × 1.00 × 1.00 mm^3^ using a linear interpolation algorithm. The signal intensities were normalized using the z-score method prior to feature extraction. A total of 1,412 radiomic features were extracted from the CE T1w and T2w images (total of 2,824). Radiomic features consist of seven classes: shape, first-order statistics, gray-level co-occurrence matrix [GLCM), gray-level size zone matrix [GLSZM], gray-level run length matrix [GLRLM), neighboring gray tone difference matrix [NGTDM], and gray-level dependence matrix [GLDM]. In addition, wavelet transform filtering was applied for textural feature extraction. Regarding wavelet filtering, the built-in stationary wavelet transformation was used through a high band-pass or lower band-pass filter in the X, Y, and Z directions, which created eight different preprocessed images, as the shape of the radiomic features could only be extracted from the original images. To test intraclass reproducibility, the data for 30 randomly selected patients were segmented twice by a single radiologist (YB) within 1 month. To test the interclass reproducibility, the same 30 sets of data were segmented by two radiologists (YB and WR). Spearman’s correlation analysis was used to assess the differences between the features generated at different times by different radiologists and between the features generated twice by the same radiologist. Interclass and intraclass correlation coefficients (ICCs) were used to evaluate the intra- and inter-observer agreement of feature extraction, where an ICC value greater than 0.80 indicated good agreement. Consequently, 2,145 features (CE T1w and T2w) were retained for further analysis. The dataset was randomly divided into training, testing, and validation cohorts from centers 1 and 2.

### Feature selection and predictive models building

**Figure 2:**
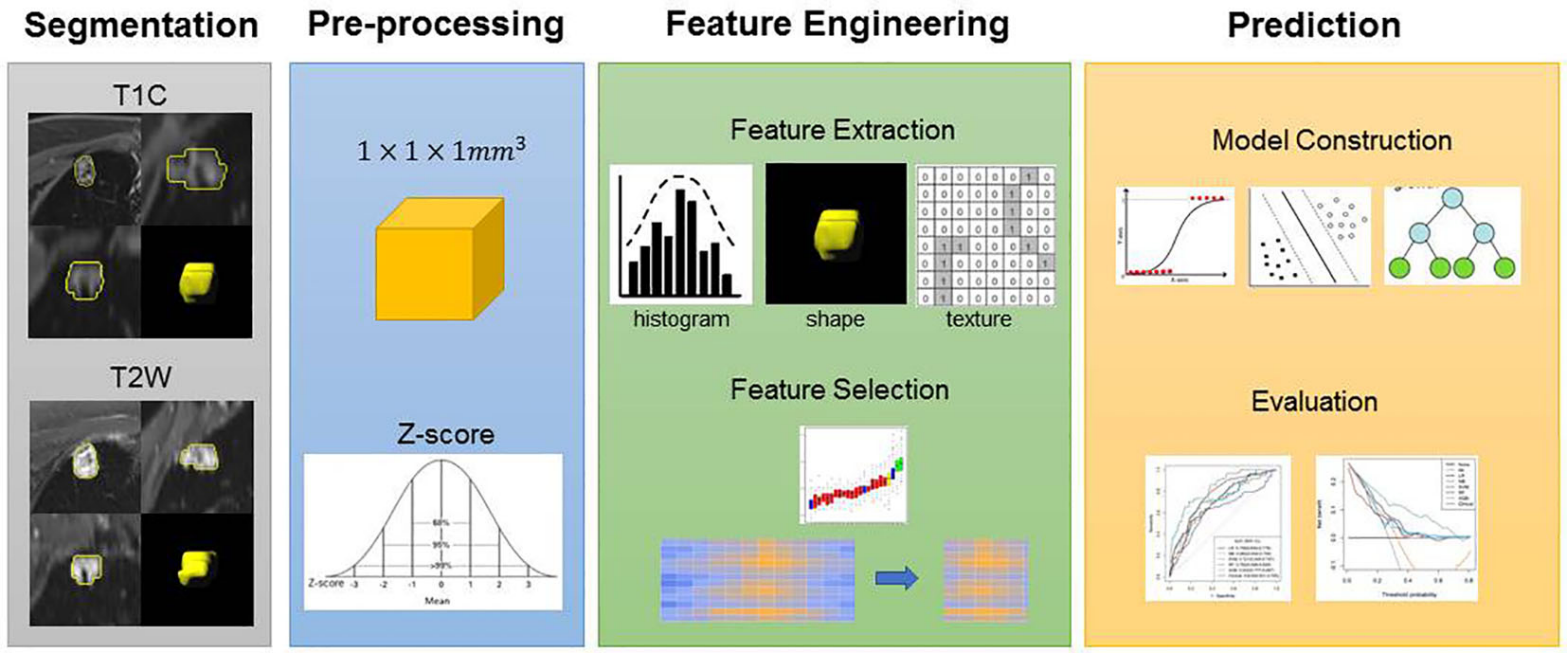
Flowchart of the experimental steps. An experienced radiologist segmented the regions of interest (ROI) of the lesions. Features were selected to build the models. Receiver operating characteristic (ROC) curves were used to demonstrate the diagnostic efficiency of the models. Decision curves were used to evaluate the potential net clinical benefit of the Prediction Models.

We used a two-step feature selection procedure to gradually select optimal features. First, univariate analysis (Mann–Whitney U test) was used to select the most useful features from the primary dataset. Second, the Boruta algorithm with threefold cross-validation for a total of 100 iterations was used. The Boruta algorithm selects a unique collection of attributes each time (25, 26). All feature selection procedures were performed in the primary cohort and applied to the final class in the validation cohort. After feature selection, five machine learning classifiers are used for building predictive models, including logistic regression (LR), Naïve Bayes (NB), support vector machine (SVM), random forest (RF), and extreme gradient boosting (XGBoost). A radiomics score (Rad-score) was obtained for each patient after applying the best machine learning model output score (**Figure 2**). A nomogram was constructed based on multivariate logistic regression analysis. Clinical factors and Rad-scores were included in the nomogram model. A calibration curve is plotted to determine the predictive accuracy of the proposed model. Decision curve analysis (DCA) was performed to evaluate clinical usefulness by quantifying the net benefits of the nomogram model.

### Statistical analysis

All statistical analyses were performed using the R software (version 4.2.0; http://www.rproject.org). Qualitative data are expressed as mean ± standard deviation (STD) for continuous data and number of cases and percentages (n [%]) for categorical data. An independent sample t test was used to compare the values between the two groups. The χ^2^ test was used to compare categorical data between the two groups. Statistical significance was set at P <0.05. The classification performance of the proposed models was evaluated using the area under the curve (AUC) of the receiver operating characteristic (ROC) curve in both the training and validation cohorts.

## Results

### Clinical characteristics of patients

A total of 333 patients were enrolled in this study: 233 (70%) were assigned to the training cohort, 100 (30%) to the test cohort, and 26 were prospectively included as the validation cohort. The clinical characteristics of patients in the training, test, and validation cohorts are shown in **Table 1**. Furthermore, 64 (27.5%) patients in the training cohort, 28 (28.0%) patients in the test cohort, and 10 (38.5%) patients in the validation cohort had malignant pulmonary nodules. There was no significant difference in the number of patients with malignant tumors among the three cohorts (P = 0.496).

### Important radiomic features selection and machine learning classifiers for building predictive models

**Figure 3:**
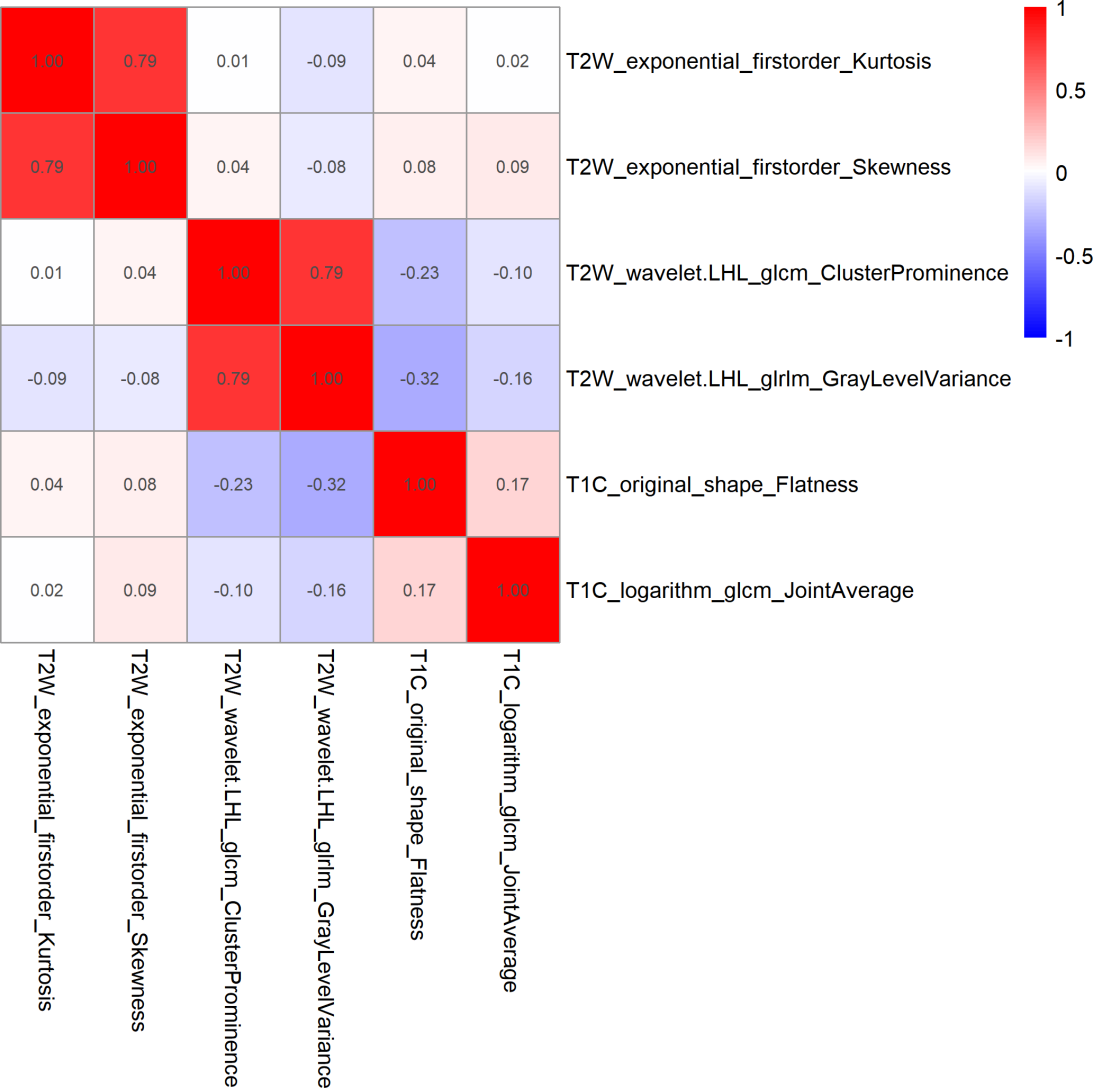
Heatmap of Spearman correlations between two of the six features that were retained. The results showed that the correlation coefficients between the two pairs of features were less than 0.9.

**Figure 4:**
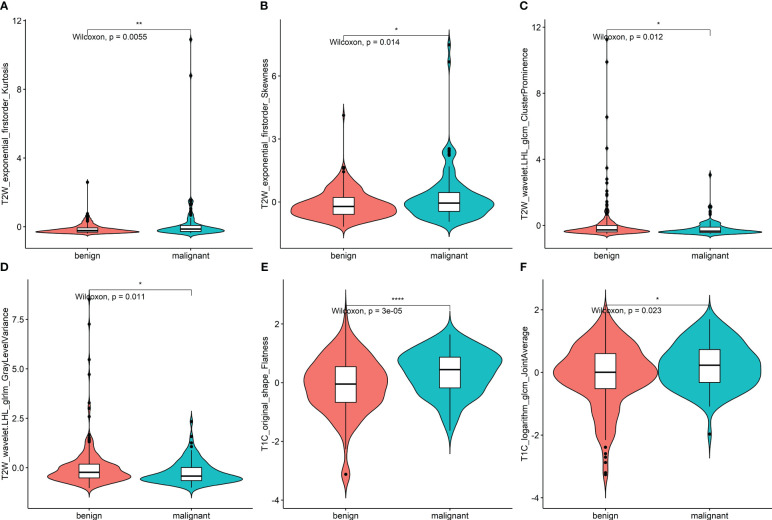
**(A–F)** Violin plot of data distribution of the remaining six features in the two groups of benign and malignant nodules after feature selection. * <0.05, ** <0.01, *** <0.001.

**Figure 5:**
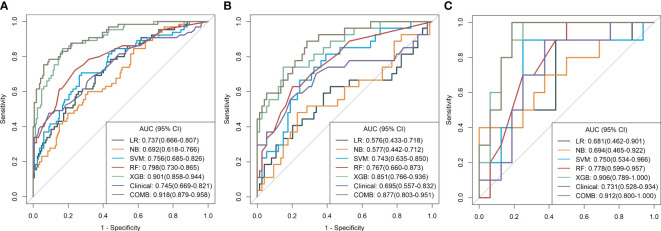
(**A–C**) Diagnostic performance of machine learning models for classifying malignant and benign pulmonary nodules based on MR radiomic features in the training, validation, and test cohorts. The number adjacent to each machine learning model is the area under the receiver operating characteristic curve. LR, logistic regression; NB, Naïve Bayes; SVM, support vector machine; RF, random forest; XGBoost, extreme gradient boosting; Clinical, clinical model; COMB, combined model.

After feature selection, the following six important features were selected from 2,145 radiomic features: T2W_exponential_firstorder_Kurtosis,T2W_exponential_firstorder_Skewness,T2W_wavelet.LHL_glcm_ClusterProminence,T2W_wavelet.LHL_glrlm_GrayLevelVariance,T1C_original_shape_Flatness,and T1C_logarithm_glcm_JointAverage (**Figures 3**, **4A–F**). The results of the Boruta algorithm selection are shown in **Figure S1**. Five machine learning classifiers were used to build predictive models: LR, NB, SVM, RF, and XGB. The AUCs of machine learning (ML) models are listed in **Table 2**. Among the five ML models, LR was 0.737, 0.681, and 0.576 in the training, validation, and test cohorts, respectively; NB were 0.692, 0.694, and 0.577 in the training, validation, and test cohorts, respectively; SVM was 0.756, 0.750, and 0.743 in the training, validation, and test cohorts, respectively; RF was 0.798, 0.778, and 0.767 in the training, validation, and test cohorts, respectively; and XGB had the highest classification performance (0.901, 0.906, and 0.851, respectively) (**Figures 5A–C**). A Rad-score was obtained for each patient after applying the best machine-learning model.

**Table 2:** Predictive performance of different classification models.

Method in cohort	AUC	ACC	SEN	SPE	PPV	NPV	cutoff
Training							
LR	0.737 (0.666–0.807)	0.634	0.769	0.581	0.417	0.866	0.256
NB	0.692 (0.618–0.766)	0.664	0.600	0.689	0.429	0.816	0.299
SVM	0.756 (0.685–0.826)	0.720	0.708	0.725	0.500	0.864	0.265
RF	0.798 (0.730–0.865)	0.750	0.723	0.760	0.540	0.876	0.130
XGB	0.901 (0.858–0.944)	0.836	0.846	0.832	0.663	0.933	0.331
Clinical	0.745 (0.669–0.821)	0.789	0.477	0.910	0.674	0.817	
COMB	0.918 (0.879–0.958)	0.875	0.785	0.910	0.773	0.916	
Validation							
LR	0.681 (0.462–0.901)	0.654	0.800	0.562	0.533	0.818	
NB	0.694 (0.465–0.922)	0.654	0.600	0.688	0.545	0.733	
SVM	0.750 (0.534–0.966)	0.731	0.700	0.750	0.636	0.800	
RF	0.778 (0.599–0.957)	0.731	0.700	0.750	0.636	0.800	
XGB	0.906 (0.789–1.000)	0.769	0.600	0.875	0.750	0.778	
Clinical	0.731 (0.528–0.934)	0.654	0.400	0.812	0.571	0.684	
COMB	0.912 (0.800–1.000)	0.808	0.700	0.875	0.778	0.824	
Test							
LR	0.576 (0.433–0.718)	0.515	0.630	0.473	0.304	0.778	
NB	0.577 (0.442–0.712)	0.594	0.519	0.622	0.333	0.780	
SVM	0.743 (0.635–0.850)	0.653	0.778	0.608	0.420	0.882	
RF	0.767 (0.660–0.873)	0.752	0.519	0.838	0.538	0.827	
XGB	0.851 (0.766–0.936)	0.822	0.741	0.851	0.645	0.900	
Clinical	0.695 (0.557–0.832)	0.772	0.296	0.946	0.667	0.787	
COMB	0.877 (0.803–0.951)	0.822	0.593	0.905	0.696	0.859	

AUC, area under the curve; ACC, accuracy; SEN, sensitivity; SPE, specificity; PPV, positive predictive value; NPV, negative predictive value; LR, logistic regression; NB, Naïve Bayes; SVM, support vector machine; RF, random forest; XGB, extreme gradient boosting; COMB, combined model.

### Development of a radiomics nomogram model

**Figure 6:**
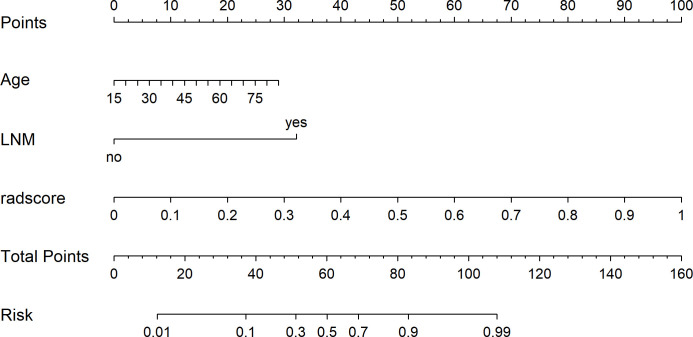
The radiomics nomogram was developed using independent predictors (rad score, age, and LNM) to predict benign and malignant pulmonary nodules.

**Figure 7:**
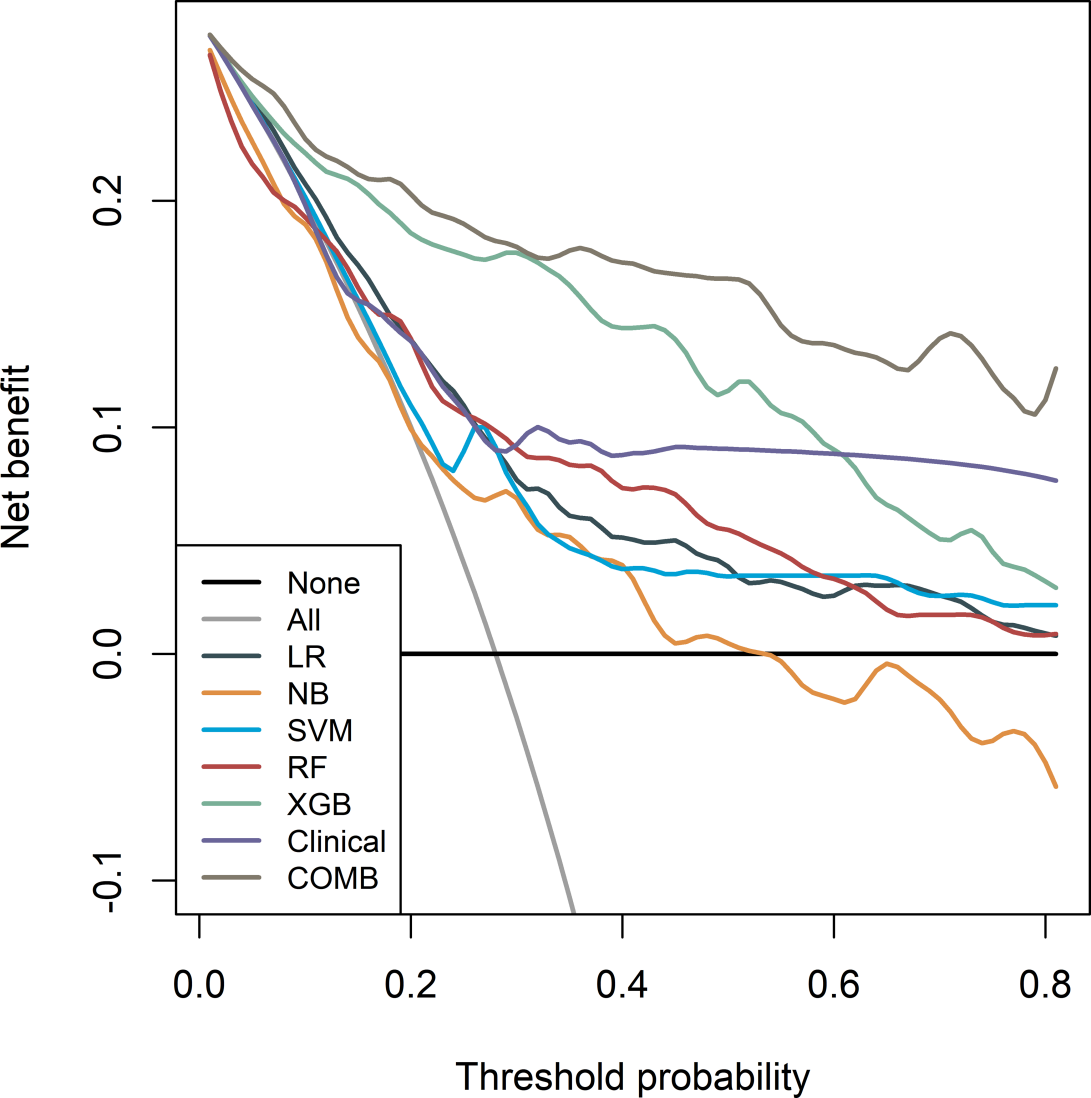
Decision curves of the seven models in the training cohort. Net income is shown on the y-axis, and the probability threshold is shown on the x-axis.

Before constructing the clinical model, clinicopathological factors were analyzed using univariate logistic regression. The predictors with P <0.05 were included in the univariate logistic regression analysis to find significant predictors. Logistic regression analysis identified age and lymph node metastasis (LNM) as independent predictors. Independent predictors (age and LNM) were used to build the clinical model with AUCs of 0.745, 0.731, and 0.695 in the training, validation, and test cohorts, respectively. Based on multivariate logistic regression analysis, the independent predictors (Rad-score, age, and LNM) were used to build the combined model and presented as a radiomics nomogram. After 10-fold cross-validation, the radiomics nomogram demonstrated AUCs of 0.918, 0.912, and 0.877 in the training, validation, and test cohorts, respectively (**Table 2**; **Figure 6**). DCA was performed to determine the clinical utility of the combined model. DCA showed that the combined model had a higher overall net benefit than the other six other clinical models (LR, NB, SVM, RF, XGBoost, and the clinical model) across most of the range of reasonable threshold probabilities (**Figure 7**).

## Discussion

In this study, five machine learning classifiers were built based on multimodal MRI to extract radiomics features. Multivariate logistic regression analysis of clinicopathologic factors was performed to screen out independent predictors, and a clinical prediction model was established to classify benign and malignant pulmonary nodules. The classification efficiency of five machine learning classifiers was compared, and the classifier with the highest prediction efficiency among the machine learning classifiers was output with the Rad-score. Finally, the Rad-score and independent predictive factors were used to establish a multiple regression model and were presented in the nomogram. The results showed that all five machine learning classifiers could be used to classify benign and malignant pulmonary nodules, and that the XGBoost classifier had the highest classification performance. The AUC for the training, validation, and test sets were 0.901, 0.906, and 0.851, respectively. The Rad-score combined with independent predictors to construct the joint prediction model showed good classification efficiency, and its AUC in the training, validation, and test sets were 0.918, 0.912, and 0.877, respectively. This model could be used to classify benign and malignant pulmonary nodules.

The nature of small pulmonary nodules is still difficult to decipher, which directly affects the choice of treatment and prognosis of patients. Differentiation between benign and malignant small pulmonary nodules less than 2 cm in diameter, avoiding unnecessary invasive examination and surgical trauma, and preventing tumor progression due to follow-up are necessary; at present, it is a hot spot for doctors in the imaging, thoracic surgery, and respiratory departments to treat these types of lung cancer patients in time and effectively. Therefore, identifying and screening more comprehensive and effective indices of lesion heterogeneity and microenvironment characteristics by non-invasive examination to achieve accurate early screening of malignant small pulmonary nodules is a key scientific problem that needs to be urgently addressed in this research. With the emergence of artificial intelligence technologies, including deep learning feature extraction and segmentation technology, deep survival analysis, and radiomics analysis, the depth features of small pulmonary nodules (<2 cm) can be extracted quantitatively and segmented accurately. The survival analysis model can be built by simulating the nonlinear risk score function, and high-throughput features in the images can be extracted. Quantitative characterization of the heterogeneity of lesions to achieve accurate prediction of disease recurrence, non-invasive prognosis, and comprehensive quantification of the heterogeneity and microenvironment of the lesions will provide a direction for the deep mining and application of medical images and will help select more appropriate, effective, and personalized treatment programs. However, currently, radiomics and deep learning techniques used in lung disease research are mostly focused on CT or PET/CT images, and guidance based on multimodal MRI radiomics for the differentiation of benign and malignant pulmonary nodules (<2 cm) to achieve personalized treatment is still rare, particularly for the design of multimodal MRI data models, fusion methods, and other issues that require further study.

MRI is useful for distinguishing between tissues with different pathological features. At the same time, DWI, IVIM, DKI, and DCE-MRI can provide more sensitive quantitative and qualitative imaging markers, which are helpful for the diagnosis, histological classification, evaluation of therapeutic effect, and prognosis prediction of small pulmonary nodules to guide clinical treatment. Satoh et al. (12) scored the DWI signal intensity of 54 pulmonary nodules in 51 patients on a 5-point scale to calculate the difference in scores between malignant and benign nodules. The results showed that the mean score of malignant nodules was significantly higher than that of benign nodules on DWI, with an ACU value of 0.796 and a threshold value of three points, with a sensitivity, specificity, and accuracy of 88.9%, 61.1%, and 79.6%, respectively. Kono et al. (27) retrospectively analyzed the dynamic contrast-enhanced features of 202 solitary pulmonary nodule of 1–3 cm in diameter. The maximum enhancement ratio, time at maximum enhancement ratio, slope of time–enhancement ratio curves, and washout ratio were assessed. In lung cancers, the time at maximum enhancement ratio was 4 min less. For all benign lesions, the time at the maximum enhancement ratio was greater than 4 min or gradual enhancement occurred without a peak time. Lung cancers have different maximum enhancement ratios and slopes than benign lesions do. Dynamic contrast-enhanced MRI is helpful for differentiating benign from malignant solitary pulmonary nodules. The absence of significant enhancement is a strong predictor of benign lesions. Feng et al. (20) tested the performance of free-breathing DCE-MRI using a radial volumetric interpolated breath-hold examination (VIBE) sequence combined with DWI for quantitative solitary pulmonary nodule (SPN) assessment. Quantitative enhancement parameters (Ktrans, Kep, and Ve) and ADC values were measured in 67 patients with pulmonary nodules who underwent conventional MRI, DWI, and dynamic contrast-enhanced MRI. The results showed that the KTRANS and Kep values of malignant nodules were higher than those of benign nodules, whereas the ADC values were lower than those of benign nodules. Using an ADC value of 0.98 × 10^−3^ mm^2^/s as a threshold, the specificity and sensitivity for the diagnosis of benign and malignant nodules were 90.6% and 80%, respectively. The results showed that high-temporal-resolution DCE-MRI using the r-VIBE technique in combination with DWI could contribute to pulmonary nodule analysis and may serve as a potential alternative to distinguish malignant from benign nodules. The above research shows that MRI multi-sequence imaging can be used as a non-invasive means to objectively and scientifically describe the morphological features of pulmonary nodules and the internal structure of the tumor. The fusion of multi-sequence MRI information is expected to replace biomarkers in the early, sensitive, and the accurate diagnosis of pulmonary nodules, thus improving the diagnostic accuracy of pulmonary solid nodules and the survival rate of patients with lung cancer, providing technical support for accurate diagnosis and treatment of pulmonary nodules. However, the current study had a small sample size, poor diagnostic efficacy, no external multicenter validation, and a lack of reliability and stability.

Therefore, in this study, we established five machine learning classifiers, one clinical predictive model, and one joint predictive model, based on MRI radiomics features. Our results show that both the machine learning classifier and joint prediction model have a high prediction efficiency. Among the machine learning classifiers, XGBoost had the highest classification efficiency, with AUCs of 0.901, 0.906, and 0.851 for the training, validation, and test sets, respectively. The XGBoost output score combined with independent predictors built a joint model with AUC of 0.918, 0.912, and 0.877 for the training, validation, and test sets, respectively. This shows that our model has better predictive power than previous studies (24, 28, 29). This may be because our machine learning classifier uses the XGBoost algorithm. XGBoost performs well in data analysis and prediction. It is a decision-tree-based integration algorithm that strives to maximize the speed and efficiency and prevent model overfitting. Combined with the Rad-score, the classification performance of the model significantly improved (30).

Regarding the advantages of our study, first, we used the XGBoost algorithm. The XGBoost model trains extremely quickly, computes the importance of each feature for feature selection, model interpretability, model transparency, and model tuning, saves the tree model in clear text, and facilitates model visualization and tuning. The XGBoost gradient promotion algorithm uses a gradient descent algorithm to minimize the prediction error and generates a set of weak prediction models (decision tree). During training, a new regression tree was added to the gradient elevation each time to reduce the residuals (the difference between the model predictions and label values). The existing trees in the model remain unchanged, which reduces the overfitting rate (31). Second, all procedures in this study were performed using the same MRI device with the standard protocol, which avoided the heterogeneity of image impressions from different scan and reconstruction parameters, thus leading to more stable and reliable results. Moreover, semiautomatic segmentation tools were implemented in our radiomics research prototype; therefore, individual differences in manual drawings were limited. Third, repeated cross-validation was used for training to reduce biased estimations, and testing was performed in a validation cohort and a prospective cohort to evaluate the performance of our model. Therefore, our model is robust and reliable.

Similar to most previous studies, our study had several limitations. First, as this was a retrospective study, there may have been selection bias; therefore, more in-depth research with a larger sample size is still needed. Second, the data used for modeling in this study were obtained from a central study with no external verification. In the next step, we will gradually conduct external verification to verify the generalizability of the model, and no corresponding standard will be available for the randomness of high-throughput features, which are highly dependent on random changes in images and imaging parameters. This suggests that a standard is required to ensure reproducibility and reliability of the study results (32).

In conclusion, our study demonstrates the ability of MRI radiomic features to differentiate malignant from benign lung nodules. We have also shown improved MRI radiomics performance with the addition of age and LNM and demonstrated the strong diagnostic performance of four commonly used ML algorithms for nodule classification, with XGBoost having the highest performance.

## Data availability statement

Data available on request due to privacy/ethical restrictions.

## Ethics statement

This study was approved by the Institutional Review Board of the First Affiliated Hospital of Nanjing Medical University (2022-NT-11, Nanjing, China) and conducted in accordance with the ethical standards of the 1964 Helsinki Declaration and its later amendments. The requirement for informed consent was waived, as protected health information (PHI) was not revealed.

## Author contributions

BY conceived the idea of the study. BY, YG, JL, YW, RW, and JS collected the data. HX and FW performed image analysis. BY wrote the manuscript. JR performed the statistical analysis. HX and FW edited and reviewed the manuscript. All the authors discussed the results and commented on the manuscript. All authors contributed to the article and approved the submitted version.
