# Supplementary material for: Quantitative analysis of chest MRI images for benign malignant diagnosis of pulmonary solid nodules
Source: Front Oncol. 2023 Aug 4;13:1212608. doi: 10.3389/fonc.2023.1212608 (PMC10436991; doi:10.3389/fonc.2023.1212608)
Supplement: Supplementary file 1 [file DataSheet_1.docx]

Table S1. Clinical characteristics of enrolled patients

| Characteristics | Training Cohort | Validation Cohort | Test Cohort | *p*- value |
| --- | --- | --- | --- | --- |
|  | *N=64* | *N=28* | *N=10* |  |
| Pathological subtype |  |  |  | 0.319 |
| Adenocarcinoma | 43 (67.1875%) | 18 (64.286%) | 7 (70.000%) |  |
| Squamous cell carcinoma | 3 (4.6875%) | 1 (3.571%) | 2 (20.000%) |  |
| NOS | 18 (28.125%) | 9 (32.143%) | 1 (10.000%) |  |

NOS, not otherwise specified


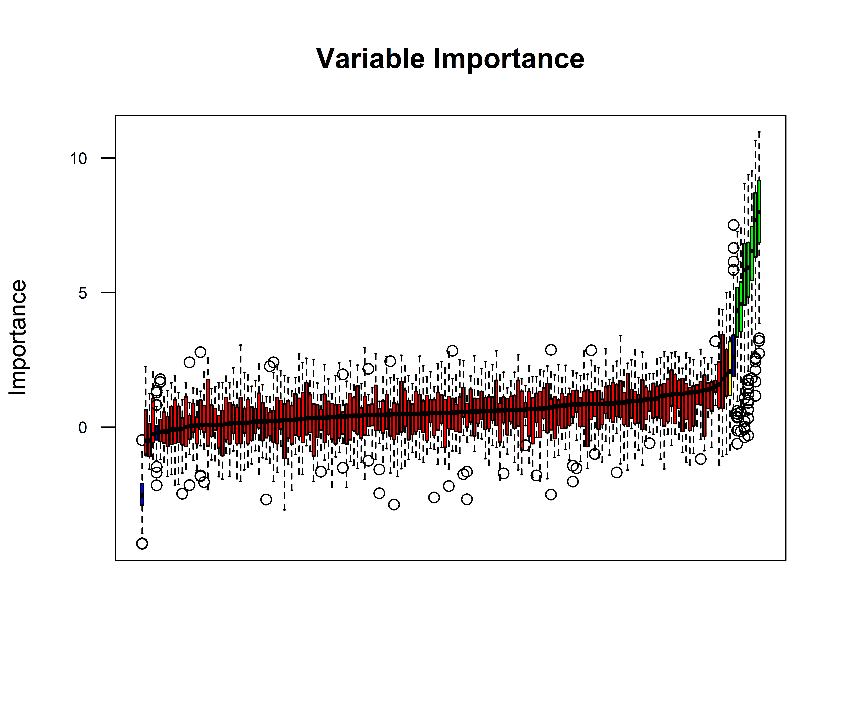


Figure S1. boxplots of attribute importance over run from Boruta algrorithm. The Boruta algorithm assesses the relevance of each feature by comparing it to a set of random shadow features, determining if it is better than the shadow features in terms of importance.
